# Case Report: Wound Closure Acceleration in a Patient With Toxic Epidermal Necrolysis Using a Lyophilised Amniotic Membrane

**DOI:** 10.3389/fbioe.2021.649317

**Published:** 2021-04-16

**Authors:** Bretislav Lipový, Martin Hladík, Petr Štourač, Serhiy Forostyak

**Affiliations:** ^1^Department of Burns and Plastic Surgery, Faculty of Medicine, University Hospital Brno, Masaryk University, Brno, Czechia; ^2^Central European Institute of Technology, Brno University of Technology, Brno, Czechia; ^3^Department of Paediatric Anaesthesiology and Intensive Care Medicine, Faculty of Medicine, University Hospital Brno, Masaryk University, Brno, Czechia; ^4^PrimeCell Bioscience Inc., Prague, Czechia; ^5^National Tissue Centre Inc., Ostrava, Czechia

**Keywords:** toxic epidermal necrolysis, lyophilised amniotic membrane, reepithelization, infection control, toxic epidermal necrolysis, amniotic membrane

## Abstract

**Background:** Toxic epidermal necrolysis (TEN) is a rare life-threatening disease that mainly affects the skin and mucous membranes, resulting from a toxic delayed-type hypersensitivity (DTH) reaction (type IV reaction) to the presence of foreign antigens such as drugs. The clinical symptoms are caused by pathophysiological processes leading to massive apoptosis of keratinocytes in the dermo-epidermal junction. This results in the formation of a bulla and subsequent separation of the entire epidermis with the exposure of the dermis. The current approach in the local therapy of TEN prefers the use of biological dressings, which helps provide several critical requirements for defect healing; in particular, it helps in the acceleration of the spontaneous wound closure (re-epithelialization) of the skin defect and the reduction of the risk of development of various complications and infections, such as the risk of pathological scar maturation. This paper is a case report of the use of a lyophilized amniotic membrane (AM) for accelerating wound healing in a patient with TEN.

**Case Presentation:** We report a case of an 8-year-old girl transferred to our center with a histologically confirmed diagnosis of TEN. Despite the application of immunosuppressive therapy consisting of corticosteroids and intravenous immunoglobulins, we have observed disease progression and exfoliation of up to 60% of the total body surface area (TBSA). In the facial area, which is cosmetically privileged, we decided to use the lyophilized amniotic membrane (Amnioderm®) to cover up approximately 2% of the TBSA. Within 2 days after the application, we observed accelerated reepithelialisation, with rapid wound closure. We have not observed any side effects nor infections during the subsequent phases of wound healing. Skin defects in non-facial areas of the body were treated with synthetic dressings. When compared to the areas covered with the lyophilized AM, the healing process was prolonged.

**Conclusions:** To our knowledge, this is the first case study using a lyophilized amniotic membrane in the treatment of a patient with TEN. The AM application in the cosmetically-privileged area (face), proved to be very efficient in the treatment of TEN patients. The use of this allogeneic material demonstrated excellent biocompatibility and caused a unique acceleration of epithelialization and wound healing, yielding also excellent long-term results. The current study opens broad possibilities for clinical application of the used material, the improvement of current therapy of patients with TEN and better outcomes and recovery of patients.

## Introduction

**Figure 1:**
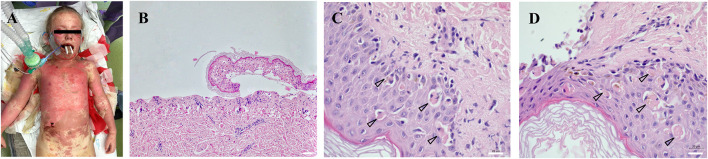
Extent and location of exfoliated areas before debridement during primary treatment in the operating theater under general anesthesia **(A)**. Partial exposure of the dermis, epidermis separated with necrosis of basal keratinocytes, which turns into complete necrosis of the entire epidermis (100x magnification) **(B)**. Apoptotic bodies (arrowheads) were visualized in the epidermis collected from the affected area at different body locations (630x magnification) **(C,D)**. All histological sections (2 × 2 mm) were prepared from formalin-fixed paraffin-embedded (FFPE) tissue specimens. Scale 20 μm.

Toxic epidermal necrolysis (TEN) is a rare, life-threatening disease predominantly manifesting on the skin and mucosa. It develops as a result of a type IV hypersensitivity reaction (delayed-type hypersensitivity, DTH), a type IVc subtype (predominantly mediated by cytotoxic T-lymphocytes). The typical clinical symptomatology occurs within a few days after the interaction of the immune system with an antigen (most commonly, a drug). The principal clinical manifestation presented by a skin exfoliation caused by apoptosis, predominantly in the dermo-epidermal junction (Figures 1C,D). Two systems participating in the development of apoptosis in TEN patients were described – the caspase and non-caspase systems. The caspase system of apoptosis is characterized by a pathway primarily activating the initiation caspase (caspase 8) and, subsequently, effector caspases (caspase 3, caspase 6 etc.) (Cecconi et al., 1998). The caspase system itself is in TEN patients triggered either through binding of a specific ligand (FasL, CD95L) to a receptor (FasR, CD95R, APO-1, TNFRSF6) or through TNFα binding to a specific receptor (TNFR1, Tumor Necrosis Factor Receptor 1) (Locksley et al., 2001; Lavrik and Krammer, 2012). For the disease to be diagnosed as TEN, exfoliation must occur on at least 30% of the TBSA (Total Body Surface Area) (Bastuji-Garin et al., 1993; Schwartz et al., 2013). If the extent is 10–30%, it is a so-called overlap TEN and if below 10%, we speak of Stevens-Johnson syndrome (SJS).

The incidence of TEN is, according to many epidemiological studies, ~0.5–1.0 cases per mil. population per year and proportionally increase with the age (Schopf et al., 1991; Ventura et al., 2010; Rodriguez-Martin et al., 2019). Age related cases occur due to the more often use of medications by older generation than the younger ones (Hsu et al., 2017). In children, TEN is rarer and in general, children have a better prognosis than adult patients. HIV patients were also shown to have a higher TEN incidence (Saiag et al., 1992). For unknown reasons, women are more frequently affected by this disease than men. The disease is also burdened with high mortality, in various studies ranging mostly between 30 and 60% (Firoz et al., 2012; Dodiuk-Gad et al., 2015; Mccullough et al., 2017).

There are many TEN classification schemes based on various evaluation parameters; one of the most widely used schemes was developed as soon as 1990s. It defines a group of severe cutaneous adverse reactions (SCARs) as a subgroup of cutaneous adverse drug reactions (CADRs) (Roujeau and Stern, 1994; Kelly et al., 1995). Three features are typical of all representatives of SCARs: (1) severity (high lethality), (2) difficult predictability, and (3) a frequent association with the use of medication. SCARs include Stevens-Johnson syndrome (SJS), TEN, DRESS (Drug Reaction with Eosinophilia and Systemic Symptoms) and AGEP (Acute Generalized Exanthematous Pustulosis).

As the pathophysiological background, the clinical development of TEN has not been fully defined yet, neither was established a standard universal systemic approach for patients treatment. Most commonly, the systemic approach includes the use of immunomodulators/immunosuppressants such as corticosteroids, cyclosporin, intravenous immunoglobulins etc. In the last decade a biological treatment has been also actively discussed (Infliximab, Etanercept) (Paradisi et al., 2014; Ganzetti et al., 2015; Chafranska et al., 2019; Zhang et al., 2020). The basic approach in complex wound-management aims protection of the exposed dermis in the region of skin exfoliation from the external physical and chemical factors to prevent the wound conversion and the loss of re-epithelization capacity. Biological dressings (temporary skin substitutes) appear to be the most suitable for these purposes and has several advantages in TEN patients – it prevents desiccation and maceration of exfoliated wounds, reduces the heat and fluid loss (thus optimizing the overall fluid management in TEN patients), forms a barrier preventing the development of infectious complications, and reduces pain. Originally, this approach involved temporary dressing of the wounds with either a porcine xenograft (Marvin et al., 1984; Heimbach et al., 1987; Schulz et al., 2000) or a cadaveric allograft (Davidson and Hunt, 1981; Spies et al., 2001).

Amniotic or amnion membrane (AM) offers an alternative for wound management utilizing other biological dressings. AM is the innermost, multilayered part of the placenta (its thickness is 0.02–0.5 mm) contributing to the homeostasis of the amniotic fluid during pregnancy. After the labor, all perinatal tissues are considered as biological waste. However, the unique composition, immunological and regenerative properties of AM make it a valuable tissue for the treatment of various wounds and regenerative medicine in general. The positive effects of amniotic membrane on the acceleration of wound healing and improving the healing quality were reported almost one hundred years ago. More recently, AMs have been shown to promote epithelialization and neovascularization, to exhibit antimicrobial effects, to reduce inflammation and fibrosis, to provide a substrate for cell growth, and to act as a biological dressing (Lipovy and Forostyak, 2020). In this study, we present the first case report of the use of the lyophilized amniotic membrane (Amnioderm®) for accelerating wound healing in a patient with TEN.

## Case Presentation

Current work aims to present a case of an 8-years old girl with a history of chemosis of the conjunctiva with fevers. These symptoms appeared from full health and were followed by maculopapular exanthema the next day, which progressed to a vesicular exanthema. Moreover, enanthema of the oral cavity appeared, together with the elevation of inflammatory markers with thrombocytopenia. The patient's medical history showed that patient underwent adenotomy and an even of bronchitis of streptococcal origin (*Streptococcus pneumoniae*) within 1 year (2014). Except for the above two conditions, the patient had been healthy, without a record of idiopathic or any known allergies, as well as without a history of prolonged use of medications. Anamnesis did not reveal any history of severe medical conditions in the family. No aetiological association between the development of the disease and the use of any drugs according to the ALDEN criteria was found. The repeated serological examination did not detect any antibodies against infectious agents (herpes simplex virus 1,2, varicella-zoster virus, cytomegalovirus, parvovirus, *Mycoplasma pneumoniae, Chlamydia pneumoniae, Legionella pneumophila*, HIV 1,2, hepatitis viruses).

After the hospitalization, an exfoliative disease was suspected and corticosteroid therapy initiated immediately. Highly suspicious clinical signs of TEN (Figures 1A,C,D) were confirmed by biopsy (Figure 1B). Despite the corticosteroid and (newly initiated) intravenous immunoglobulin (IVIG) therapy, the disease progressed and the patient was transferred on Day 8 from the Pediatric Intensive Care Unit of the local hospital to the Burn Intensive Care Unit (BICU) of the University Hospital Brno. The BICU is a specialized center with experience and expertise in the management and surgical therapy of TEN patients who opted for this type of treatment. On admission to the BICU, the patient was breathing spontaneously. Sixty per cent of the Total Body Surface Area (TBSA) was exfoliated (face, neck, thorax, upper extremities and proximal parts of lower extremities; Figure 1A). Moreover, a symblepharon of both anterior segments of the eyeball was found. Due to the relatively high damage to the mucosal surfaces of the upper respiratory tract, the patient needed intubation during the introduction to general anesthesia, along with the placement of central venous and permanent urinary catheters. Bronchoscopy revealed a fragile and bleeding mucosa; therefore, the decision was made to keep the patient intubated. After the surgery, a girl was transferred to the Department of Pediatric Anaesthesiology and Resuscitation of the University Hospital Brno (PARU) for further intensive care.

### Systemic Therapy

In the PARU the patient was administered intravenous methyl-prednisolone (starting on Day 5, 80-40-40 mg every 8 h), antimicrobial therapy consisting of oxacillin (800 mg every 6 h), cefotaxime (1.5 g every 8 h) and, due to the increase of ß-D-glucan level (155 pg/ml), fluconazole (150 mg every 24 h) was added into the therapy. The IVIG therapy continued (0.2 g/kg/day, 5 days administration/25 g cumulative dose) and was supplemented with cyclosporin (2.5 mg/kg/day divided into 2 doses, 5 days administration). Immunological blood tests showed a significant decrease of CD3^+^ lymphocytes (in subpopulations of CD4^+^ and CD8^+^) but no immunoparalysis was proven (expression of CD14^+^ at monocytes was around 100%).

Despite the above therapy, a patient has developed delirium, which complicated extubation (microaspiration) and caused a need for a re-intubation (9th day of hospitalization). Considering the above and perspective of long-term mechanical ventilation, surgical tracheostomy was performed (11th day of hospitalization). After the patient's condition improved, mechanical ventilation was discontinued without any problems and she was transferred back to the Paediatric Intensive Care Unit of the local hospital for further alimentation and rehabilitation (19th day of hospitalization).

## Local Therapy

**Figure 2:**
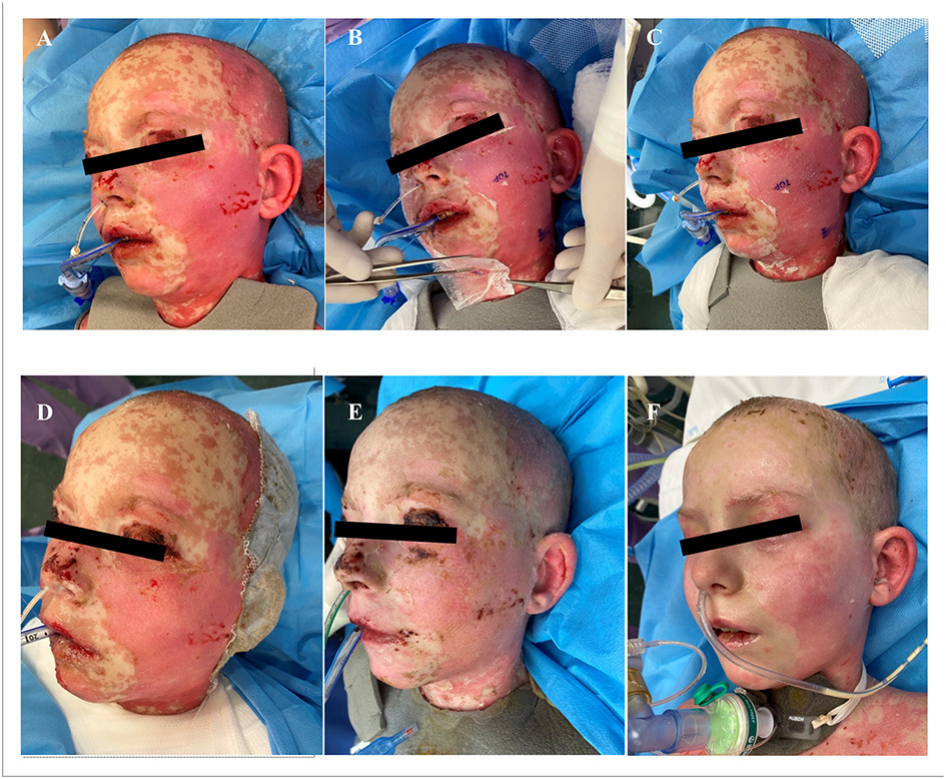
Facial areas after debridement before application of lyophilized amniotic membrane **(A)**, application of lyophilized amniotic membrane **(B,C)**. Subsequent re-epithelialization in the facial area, 48 h after application of the lyophilized amniotic membrane **(D)**, 7 days after application **(E)**, 14 days after application **(F)**.

**Figure 3:**
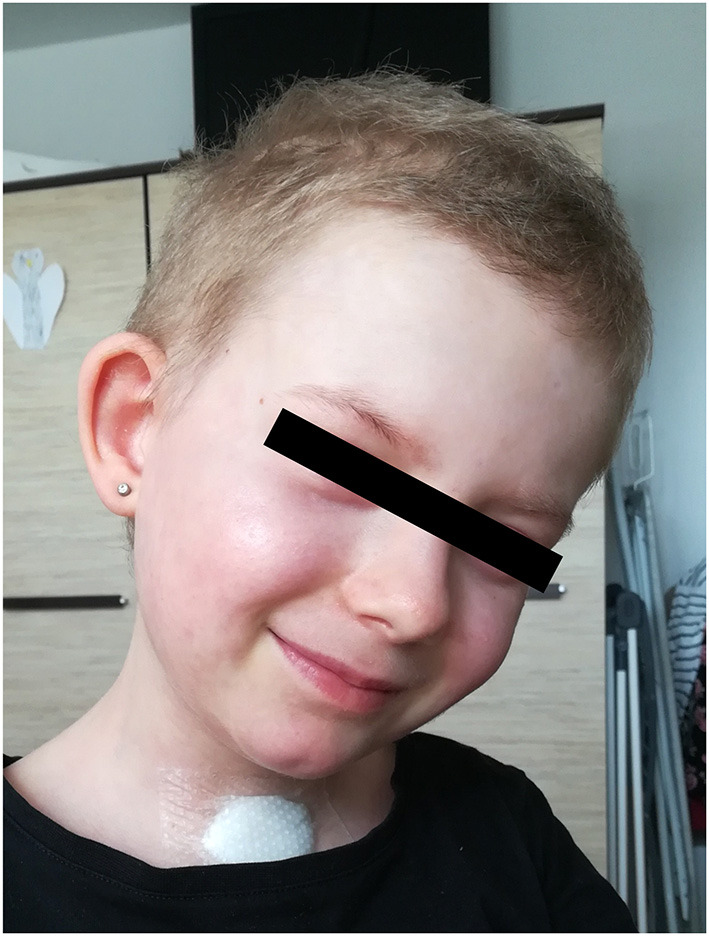
Local image 3 months after application of lyophilized amniotic membrane - no signs of hyperpigmentation.

Additionally to the systemic therapy, the patient received specialized surgical management of the vast skin defects, comprising of the surgical debridement, wound dressing and stimulation of regeneration. We have decided to us a unique combination of synthetic (cosmetically nonprivileged areas) and biological (cosmetically privileged area) dressings in addition to the standard of wound care. For the very first time as a part of TEN therapy, we have used as a biological dressing a novel material - lyophilized amniotic membrane (Amnioderm®). Amnioderm® was used in the facial area. The remaining exfoliated skin defects were covered using a combination of synthetic dressings (Aquacel Ag + Extra®, ConvaTec, Princeton, NJ, USA and Mepilex Ag®, MölnlyckeHealth Care AB, Gothenburg, Sweden). The bandages were replaced 3 times a week under general anesthesia. After the application of Amnioderm®, we observed a rapid re-epithelization within 48 h (Figure 2). All skin defects were healed by re-epithelization within 3 weeks after admission to the BICU. We observed no scar formation in the area of AM application. During the 3 months follow-up period, we observed a total regeneration of the skin with no signs of pigmentation or scaring at the area of Amnioderm® application (Figure 3). The area treated with the synthetic dressings was regenerating slower. Compared to the area where AM was applied, healing was extended by 4–5 days to complete wound closure. During the following period, post-exercise hyperaemia without and signs of hyperpigmentation persisted in the areas treated with synthetic dressings.

## Discussion

A proper wound/exfoliated area management in TEN patients play a key role in spontaneous defect closure. The absence of robust data and the non-existence of a consensus in the complex wound management in TEN patients results in the management of the TEN wounds following the rules for the treatment of burns (Endorf et al., 2008; Creamer et al., 2016; Castillo et al., 2018). However, there are several fundamental differences between two types of wounds: the dynamics of tissue devitalization (predominantly necrosis via IL-1 pathway in burns) and apoptosis/necroptosis (mediated predominantly by TNF-α in TEN patients) (Nassif et al., 2004; Kinoshita and Saeki, 2016). In TEN patients, the healing time is greatly affected by the administration of immunosuppressants. Two approaches are applicable in early wound-management in SJS/TEN patients – conservative and interventional (surgical).

The conservative approach is often promoted in dermatological guidelines, in which leaving the necrotic epidermis *in situ* is recommended, together with a careful perforation of the bullae, which ensures sufficient contact with the wound floor. The necrotic epidermis then serves as a biological dressing capable of fluid loss reduction, reduction of heat loss, pain and, last but not least, of the risk of developing infectious complications. The main disadvantage of the conservative approach is the fact that together with the necrotic keratinocytes, the CD8^+^ T lymphocytes and other cells populations remain in the wound, as well as proinflammatory cytokines. Those can further increase the oxidative and nitrosative stress and thus interfere with wound healing. Moreover, the persisting proinflammatory condition has definite long-term adverse effects (i.e., hyperpigmentation of healed defects).

On the other hand, the surgical approach is based on a precise debridement, i.e., complete removal of the devitalized tissues. This approach is preferred in particular in SJS/TEN patients hospitalized in burn centers. The debridement itself exposes the dermis, which is very sensitive to many factors of the external environment. The exposed dermis is prone to developing infectious complications (protective barrier mechanisms are missing), higher fluid and heat loss, and greater pain. Most of these factors are capable of causing secondary necrosis in the area of the exposed dermis and to wound conversion, i.e, to the loss of re-epithelization capacity. This leads to a dramatic worsening of the patient prognosis. For this reason, the emphasis is put on the wound dressing quality.

The use of dressing material after debridement become of the utmost importance to ensure problem-free defect healing. Nowadays, a wide range of quality dressings (synthetic, biosynthetic and biological) are available. The biological dressings include allografts, xenografts, amniotic membrane, collagen sheets, or cultured epidermal autografts (CEAs) (Bhattacharya et al., 2011; Papp et al., 2018). This concept, known as the “Race for the surface,” was described by the orthopedic surgeon Anthony G. Gristina as soon as 1987 (Gristina, 1987). Biological dressings are preferred for use in patients with SJS/TEN. A relatively recent study disclosed that 38% of North American Burn Centers and Dermatology Departments use bioactive skin substitutes as a first choice in complex TEN wound management. However, relevant comparable data from other parts of the world are missing. In the Czech Republic, there is a long history and experience for the use of various biological skin transplants and dressings in the management of wound-healing, including an acellular porcine dermis (Xe-Derma®) (Lipovy et al., 2014). The principal disadvantage of biological dressings is their price. Other discussed disadvantages include poor antimicrobial control and varying degrees of adherence. Nevertheless, although biological dressings have, unlike many silver- or antibiotic-impregnated dressings, no clearly defined antimicrobial activity, the acceleration of re-epithelialization caused by these dressings represents an effective action preventing the development of infectious complications on itself (Yang et al., 2007; Huang et al., 2010; Smith et al., 2015).

Biobrane® (Smith & Nephew UK Limited, London, UK) combines the advantages of biological and synthetic membranes (Kucan, 1995; Rogers et al., 2017). This semi-synthetic, elastic, bi-laminate temporary skin substitute that was originally developed in 1979 is widely used in TEN patients. The outer layer consists of a semi-permeable silicone layer (mimicking the epidermis) and an inner layer – a woven nylon mesh imbued with Type 1 porcine collagen. The greatest advantage of this material is its good adherence to the wound floor and formation of the environment optimal for the protection of exposed dermis, potentiating keratinocyte differentiation, migration and proliferation and, thus, the exfoliated wound closure. The silicone membrane also acts as very effective protection from external microbial contamination (Arevalo and Lorente, 1999; Boorboor et al., 2008).

Synthetic dressings containing silver are as well widely used as a wound dressing material in TEN. Mainly Aquacel Ag+Extra® and Mepilex Ag®. Silver ions have a broad-spectrum of antibacterial properties binding to the cell wall of bacteria, disruption of DNA and blocking the respiratory chain causing bacterial death (Lansdown, 2002). Synthetic dressings with silver are considered a good and cost-effective alternative for the treatment of patients with TEN (adult and pediatric population). It has many advantages as good availability, adherence and ease of application (Huang et al., 2008; Mccarthy and Donovan, 2016). Furthermore, their study claims that 95% of wound healing was achieved with no need for skin grafting. No infectious complications or allergy were observed (Huang et al., 2010). Current efforts are also devoted to develop novel biomaterial formulations that can mimic the complexity of the native ECM—a concept called biomimicry—with an impact for bioprinting applications. The ideal hydrogel formulation should reach a compromise between preserving cell viability and matching optimal printability. Another approach using *in situ* bioprinting devices also considered to have a high potential to treat defects in the skin by using the endogenous surrounding tissues to integrate deposited bioinks and to regenerate damaged tissues. Especially the use of handheld devices has high potential to be translated into the clinics due to easy operations, which might be preferred by clinicians (Heinrich et al., 2019).

Although the current case report is the first to describe the application of the lyophilized AM on the exfoliated facial area, the use of this type of dressing in TEN patients is not so rare. There are relatively robust data on the use of AM in the surgical management of ocular involvement and conjunctival mucosal damage. AM has been shown to: promote epithelialization, to reduce inflammation and fibrosis, to promote neovascularization, painkilling effect and to provide a substrate for skin cell growth, and functions as a biological bandage (Sippel et al., 2001; Cirman et al., 2014). AM also contains some immunoregulatory factors, such as HLA-G and Fas ligand and well-documented re-epithelialization effects, non-tumorigenic, anti-microbial and anti-inflammatory properties (Koizumi et al., 2000; Kubo et al., 2001; Iranpour et al., 2018). The damage to the mucosa of the frontal ocular segment is quite common in acute stage TEN patients and usually manifests as an ocular inflammation with epithelial defects manifesting on the corneal and/or conjunctival epithelium (Figure 1A). Early use of AM leads to the suppression of the inflammation, subsequently supporting the epithelial defect closure, and to a reduction of the risk of long-term consequences that can dramatically reduce the quality of life of the recovered patients (dry eye, ocular surface keratinization, ankyloblepharon, entropion with trichiasis, lagophthalmos, and others) (John et al., 2002; Yang et al., 2016; Jongkhajornpong et al., 2017; Shanbhag et al., 2019). Only cryopreserved AM is being used for this purpose, there is so far no data on the lyophilized AM.

The current case report demonstrates that the application of dehydrated human amniotic membrane in TEN patients results in remarkable clinical effectiveness when compared with the standard of care. Amnioderm® has excellent handling characteristics and operational efficiency. It appears to be a clinically and economically option to be implemented as standard care for TEN patients.

## Data Availability Statement

The raw data supporting the conclusions of this article will be made available by the authors, without undue reservation.

## Ethics Statement

The studies involving human participants were reviewed and approved by the Ethics Committee for Multicentre Clinical Trials of the University Hospital Brno. ID: 02-270420/EK. Written informed consent to participate in this study was provided by the participants' legal guardian/next of kin. Written informed consent was obtained from the minor(s)' legal guardian/next of kin for the publication of any potentially identifiable images or data included in this article.

## Author Contributions

BL, MH, PŠ, and SF contributed to the preparation of this paper equally. All authors contributed to the article and approved the submitted version.

## Conflict of Interest

SF was employed by company PrimeCell Bioscience Inc. and National Tissue Centre Inc. The remaining authors declare that the research was conducted in the absence of any commercial or financial relationships that could be construed as a potential conflict of interest.
